# *Bacillus*-Based Direct-Fed Microbial Reduces the Pathogenic Synergy of a Coinfection with Salmonella enterica Serovar Choleraesuis and Porcine Reproductive and Respiratory Syndrome Virus

**DOI:** 10.1128/iai.00574-21

**Published:** 2022-03-07

**Authors:** Federico A. Zuckermann, Robert Husmann, WeiYu Chen, Patrick Roady, Janice Pfeiff, Kyle R. Leistikow, Megan Duersteler, Sona Son, Michael R. King, Nathan R. Augspurger

**Affiliations:** a Department of Pathobiology, College of Veterinary Medicine, University of Illinois at Urbana-Champaign, Urbana, Illinois, USA; b Microbial Discovery Group, Franklin, Wisconsin, USA; c United Animal Health, Sheridan, Indiana, USA; Georgia Institute of Technology School of Biological Sciences

**Keywords:** direct-fed microbial, *Salmonella*, probiotic, bacillus, pathogenicity, innate immunity, NOD2, TREM-1, disease resistance, immunopathogenesis, pathogens, porcine reproductive and respiratory syndrome virus, respiratory pathogens, synergism

## Abstract

Viral respiratory infections predispose lungs to bacterial coinfections causing a worse outcome than either infection alone. Porcine reproductive and respiratory syndrome virus (PRRSV) causes pneumonia in pigs and is often associated with bacterial coinfections. We examined the impact of providing weanling pigs a *Bacillus*-based direct-fed microbial (DFM) on the syndrome resulting from infection with either Salmonella enterica serotype Choleraesuis alone, or in combination with PRRSV. Nine days after the bacterial challenge, Salmonella was isolated from ileocecal lymph nodes of all challenged pigs regardless of DFM treatment. Compared to the single bacterial challenge, the dual challenge with Salmonella and PRRSV resulted in a pathogenic synergy exhibited by a higher rate of Salmonella colonization in the lung and a more extensive and severe interstitial pneumonia. Provision of DFM to dually challenged pigs reduced the rate of lung colonization by Salmonella, eliminated or reduced the presence of PRRSV in the lung, and reduced the extent and severity of gross lung pathology. Dually challenged pigs that received DFM had increased concentrations of interleukin 1 (IL-1) and IL-8 in lung lavage fluids, accompanied by increased expression in their blood cells of nucleotide-binding oligomerization domain receptor 2 (NOD2) and triggering receptor expressed in myeloid cells 1 (TREM-1) molecules. These changes in pulmonary inflammatory cytokine production and increased expression of NOD2 and TREM-1 suggest that the DFM exerted a systemic modulating effect on innate immunity. These observations are consistent with the notion that tonic stimulation by gut-derived microbial products can poise innate immunity to fight infections in the respiratory tract.

## INTRODUCTION

Weaning imposes major physiological stress resulting in reduced feed intake and growth (1), which combined with the presence of viral and bacterial pathogens contributes to increased morbidity and mortality. Weaning stress also impairs intestinal integrity, disturbs digestive and absorptive capacity, and increases intestinal oxidative stress and susceptibility of piglets to disease (2, 3). Therefore, efforts aimed to improve nutrient digestibility and resist disease in weaned pigs are necessary to increase their survival rate at this most vulnerable stage. A healthy gastrointestinal (GI) tract consists of several important features: a healthy proliferation of intestinal epithelial cells, an integrated gut barrier function, a preferable or balanced gut microbiota, and a well-developed intestinal mucosal immune system (4). Evidence increasingly suggests that nutritional intervention at weaning using feed additives and direct-fed microbials (DFM) is a promising approach to enhance intestinal health of pigs (5, 6), although the exact protective mechanisms may vary and are still not completely understood.

Recognition of the gut microbiome’s influence in maintaining gut homeostasis, innate immunity, and susceptibility to infectious diseases is growing (7, 8). Thus, therapeutic modulation of the microbiota by the provision of DFM (or probiotics) to prevent or treat infectious diseases is recognized as a major strategy to promote gut health and improve host immunity (9–11). For example, the colonization of germfree piglets with probiotic Escherichia coli Nissle 1917 was found to suppress clinical signs, intestinal histopathological changes, and the induction of inflammatory cytokines resulting from a Salmonella enterica serovar Typhimurium challenge (12). Among the many types of probiotics being promoted, strains of *Bacillus* are attractive as DFM supplements in animal feed due to their ability to produce resilient spores (13) and a wide variety of antimicrobial compounds (14). A mixture of *Bacillus*-based DFM was found to improve goblet cell function in the porcine gut mucosa and ameliorate enteritis resulting from an enterotoxigenic Escherichia coli challenge (15). Remarkably, emerging evidence indicates that the gut microbiota’s influence on immunity extends beyond the gastrointestinal tract and into the respiratory tract, where inflammation modulating antiviral (16) and antibacterial (17) immunity has been observed. Studies have shown that the gut microbiota is broadly protective against respiratory infections, as its depletion or absence in mice leads to impaired immune responses and worsens outcomes following bacterial or viral respiratory infection (18). In humans, a double-blind randomized controlled trial of critically ill neonates given probiotics showed a significantly lower rate of nosocomial pneumonia (18% versus 36%) than with placebo treatment (19). Similarly, a clinical study determined that a 4-week course of probiotics diminished the presence and severity of respiratory symptoms in patients afflicted with an upper respiratory infection (20).

Salmonella enterica serovar Choleraesuis is a facultative intracellular pathogen capable of infecting and replicating monocytes and macrophages (21, 22). In swine, S. Choleraesuis invades the intestinal epithelium, produces enterocolitis, and colonizes the ileocecal lymph nodes (ICLN) (23) and has the unique ability to disseminate to systemic sites, causing sepsis (24, 25). A frequently affected organ is the lung, where the blood-borne infection with Salmonella manifests as interstitial pneumonia (24, 26). Notably, in field cases, coinfection of swine with S. Choleraesuis and porcine reproductive and respiratory syndrome virus (PRRSV) has been associated with more severe disease, including septicemic salmonellosis and severe pneumonia (27). Experimentally, PRRSV has also been shown to render pigs more susceptible to S. Choleraesuis septicemia, resulting in more severe systemic and respiratory disease (28). Though several DFM are marketed to improve intestinal immunity and regulate the gut microbiota (2, 5), few *in vivo* models have been used to determine the mechanisms and impact of DFM in hosts challenged with pathogens that produce respiratory diseases. To address this gap, we examined the effect of supplementing the diet of weaner pigs with a *Bacillus*-based DFM, Provent ECL, on the clinical outcome of an experimental cochallenge of S. Choleraesuis and PRRSV.

## RESULTS

### Pathogenic synergy between S. Choleraesuis and PRRSV.

An initial trial (Table 1) was performed with weanling pigs to confirm the reported pathogenic synergy between S. Choleraesuis and PRRSV (28). Pigs that were challenged with S. Choleraesuis alone (S group) or with both S. Choleraesuis and PRRSV (SP group) exhibited mild respiratory and gastrointestinal clinical signs, characterized by mild dyspnea, depression, decreased feed consumption, decreased fecal output, and loose stool between the 5th and 7th days after bacterial challenge. Pigs challenged only with PRRSV (P group) exhibited mild dyspnea, depression, and decreased feed consumption but no gastrointestinal signs. Nine days after the Salmonella challenge, all animals were euthanized and necropsied. Salmonella was isolated from the ileocecal lymph nodes (ICLN) collected from all pigs in the S and SP groups but not from the nodes of any pig in the P group. Salmonella was also isolated from the lungs of all three pigs in the SP group but only from one of three pigs in the S group and from none of the pigs in the MP group (Table 1), suggesting an increased ability of the bacteria to colonize the lung.

**TABLE 1:** Isolation of Salmonella and extent of lung pathology in pigs enrolled in the Salmonella/PRRSV synergy trial

Pathogen challenge^*a*^	Group designation	Frequency of Salmonella-positive ileocolic lymph nodes (%)	Frequency of Salmonella-positive lungs (%)	Extent (%) of gross interstitial pneumonia (±SEM)	Frequency of lungs with consolidation (%)
Salmonella	S	3/3 (100)	1/3 (33)	70 (±3)	1/3 (33)
PRRSV	P	0/2 (0)	0/2 (0)	65 (±5)	0/2 (0)
Salmonella and PRRSV	SP	3/3 (100)	3/3 (100)	91 (±2)	3/3 (100)

a Two groups of pigs (*n* = 3) were inoculated with 10^8^ CFU of S. Choleraesuis delivered into the esophagus, followed 3 days later with an intranasal administration with either 5 × 10^4^ TCID_50_ of PRRSV (group SP) or a mock inoculum (group S). Pigs in a third group (*n* = 2) received orally a capsule with a mock inoculum and were challenged 3 days later with PRRSV (group P). Nine days after the bacterial challenge the animals were euthanized and necropsied, and their lungs were scored for gross pathology. The indicated tissues were assayed for the presence of Salmonella.

**FIG 1:**
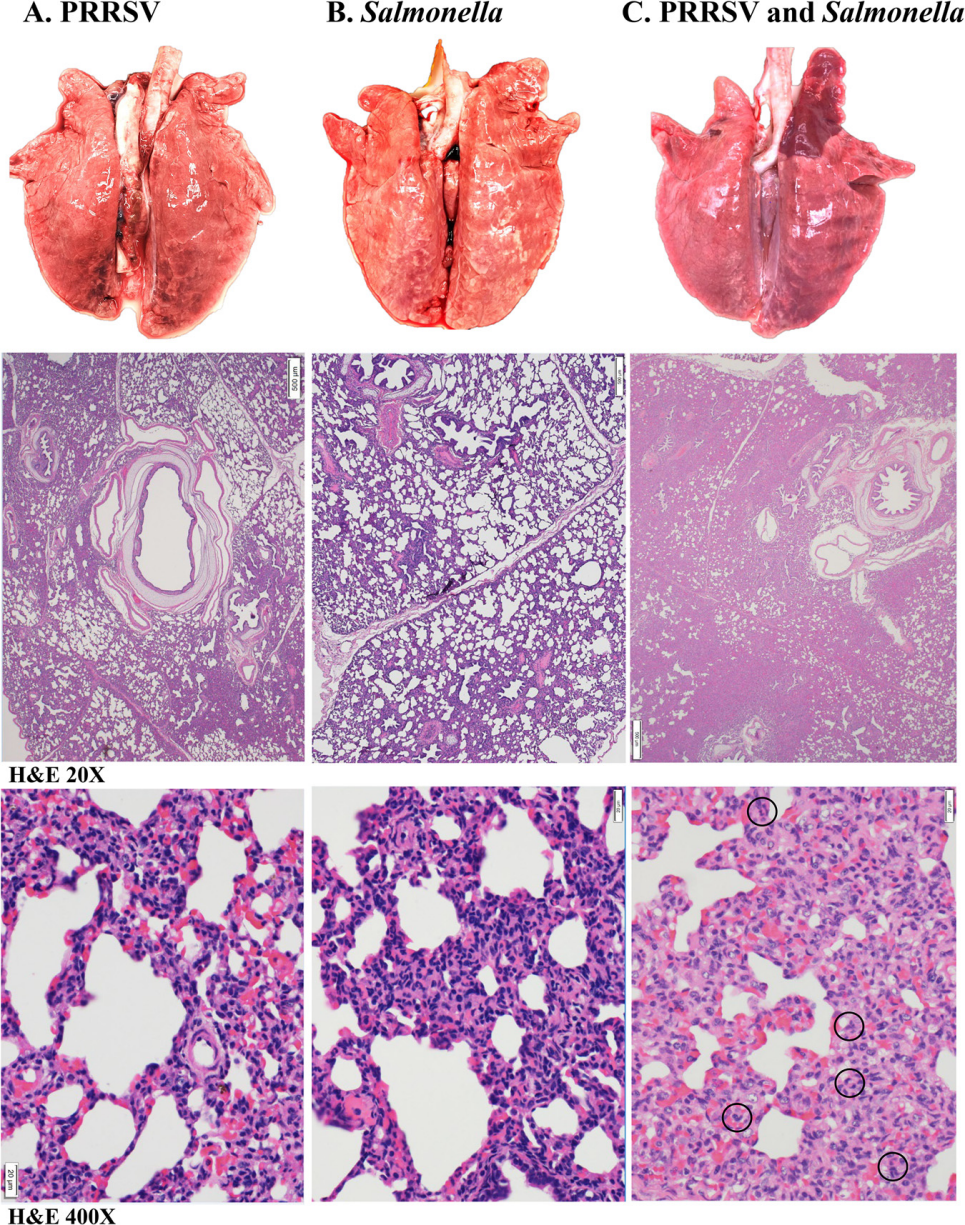
Gross and microscopic lung pathology observed in the Salmonella/PRRSV synergy trial. Shown are representative macroscopic (top row) and hematoxylin and eosin (H&E)-stained microscopic (second and third rows) images of lungs harvested at necropsy from the pigs enrolled in the synergy trial that were inoculated with either PRRSV (A), S. Choleraesuis (B), or both S. Choleraesuis and PRRSV (C). In the third row, black circles indicate the locations of neutrophils.

The most consistent pathological finding observed in the abdominal viscera of all animals challenged with Salmonella, either alone or in combination with PRRSV, was enlarged and moist ileocecal lymph nodes, enterocolitis, and the presence of randomly scattered small pale foci in the liver. This type of foci represents areas of multifocal liver necrosis that result from the septicemic spread of S. Choleraesuis (24, 29, 30). None of the animals challenged only with PRRSV displayed lesions in the liver. Gross lung lesions in pigs challenged only with PRRSV (Fig. 1A, top) or Salmonella (Fig. 1B, top) were similar in extent and type (Table 1). In both groups, the lesions observed consisted of a mild to moderate interstitial pneumonia, characterized by multifocal tan mottled areas with irregular and indistinct borders that were variably scattered without predilection for a particular lobe and affected 40% to 70% of the total lung surface area. In contrast, lungs from the pigs challenged with both Salmonella and PRRSV exhibited a more extensive and severe pneumonia (Table 1), evidenced by variably enlarged and partially collapsed lungs that felt heavy, wet, and rubbery, with randomly spread mottled areas affecting 40% to 90% of lung surface and coalescing red patchy areas with a firm liver-like consistency commonly referred to as lung consolidation (Fig. 1C, top). Pathologically, lung consolidation represents a pulmonary exudate or other product of disease that displaces alveolar air, rendering the lung tissue solid.

Histologically, the lungs of pigs challenged only with Salmonella revealed the presence of a mild interstitial pneumonia with an approximately 20% decrease of alveolar space characterized by a mild to moderate septal thickening (Fig. 1B, middle), which was infiltrated by mononuclear cells (Fig. 1B, bottom). The lungs of pigs challenged only with PRRSV showed changes consistent with a mild to moderate interstitial pneumonia, with an approximately 10% decrease of alveolar space (Fig. 1A, middle). The loss of alveolar airspace was due to a mild to moderately thickened pulmonary interstitium due to infiltration by mononuclear cells (Fig. 1A, bottom). In contrast, histological analysis of lungs collected from pigs challenged with both Salmonella and PRRSV revealed lesions consistent with severe interstitial pneumonia and lung consolidation. In this group, lung lesions were characterized by a 30 to 60% decrease of alveolar spaces (Fig. 1C, middle). The septa were variably expanded by mononuclear cells, histiocytes, and occasional neutrophils (Fig. 1C, bottom). Interlobular septa and perivascular connective tissue were mildly expanded by edema, lymphocytes, macrophages, and rare neutrophils. Thus, the gross appearance of lung consolidation was the result of a more severe interstitial pneumonia characterized by major inflammatory cell infiltration, namely, mononuclear cells and a few neutrophils, which resulted in a pronounced disappearance of alveolar air space.

### Pigs receiving DFM exhibited a reduced extent and severity of lung pathology resulting from the pathogenic synergy between S. Choleraesuis and PRRSV.

**FIG 2:**
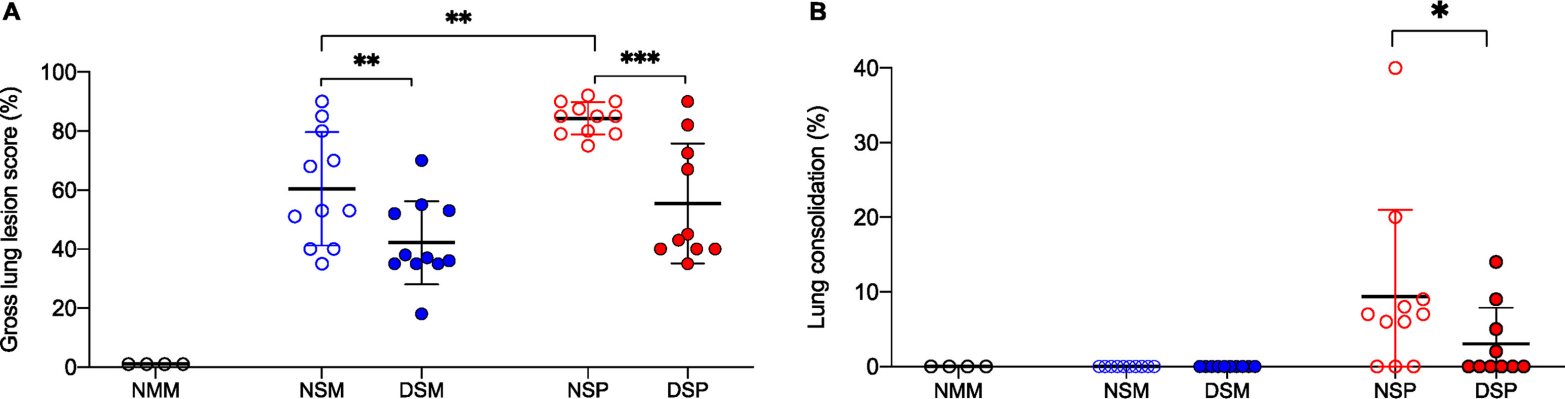
Extent of gross lung pathology and lung consolidation. Groups of weaner pigs were fed for 21 days study with a standard balanced soybean/corn swine phase 2 nursery diet, supplemented (filled circles) or not (empty circles) with direct-fed microbial (DFM). Afterwards, pigs were challenged with Salmonella enterica serotype Choleraesuis, followed 3 days later with an intranasal challenge with either PRRSV (red circles) or a mock inoculum (blue circles), while continuing to be provided the same respective diet. The control group was mock challenged and fed a nonsupplemented diet (black circles). (A) Gross lung lesion score is given as an estimate of the percentage of lung with grossly visible pneumonia. (B) Extent of lung consolidation is given as an estimate of the percentage of lung exhibiting hepatization as determined tactilely by tissue firmness. Each symbol represents the score given to each lung grouped by treatment. Each treatment group is identified by its corresponding abbreviation: non-DFM treated and mock challenged (NMM), non-DFM treated and challenged only with Salmonella (NSM), DFM treated and challenged only with Salmonella (DSM), non-DFM treated and challenged with both Salmonella and PRRSV (NSP), and DFM treated and challenged with both Salmonella and PRRSV (DSP). Horizontal bars represent the mean ± SD of each group. In panel A, differences between groups were analyzed using one-way ANOVA, followed by Tukey’s multiple comparisons. In panel B, differences between groups NSP and DSP were analyzed using Mann-Whitney *t* test. Differences were considered significant if the *P* value was <0.05. *, *P* < 0.05; **, *P* < 0.01; ***, *P* < 0.001.

The experimental design and treatment group designation for the second trial are listed in Table 2. At necropsy, examination of the abdominal cavity of all pigs challenged with Salmonella revealed that the mesenteric lymph nodes, in particular those draining the ileocecal junction, were edematous and enlarged, and evidence of enterocolitis around the ileocecal junction was observed. Pigs challenged only with Salmonella and without DFM treatment (NSM group) exhibited pathological changes typical of interstitial pneumonia involving, on average, 60% ± 6% of the lung (Fig. 2A). In contrast, the lungs of pigs challenged with both Salmonella and PRRSV, without provision of DFM (NSP group), exhibited a significant (*P* < 0.01) increase in extent of lung involvement (84% ± 1.6%). Typically, interstitial pneumonia develops in response to septicemic salmonellosis, characterized by a diffuse distribution of tan mottled changes, accompanied by petechial hemorrhages under the pleura that penetrate into the lung parenchyma. However, in the group challenged with both PRRSV and Salmonella without DFM treatment (NSP group), in addition to such lesions, 8 of 11 pigs also exhibited the presence of areas with lung consolidation (Fig. 2B) that were variably distributed in the cranioventral, middle, and caudal lobes. As described above (Fig. 1), the appearance of lung consolidation indicates a more severe pulmonary inflammatory response. In both challenge groups, provision of DFM resulted in a significant decrease in the extent of interstitial pneumonia (Fig. 2A). Remarkably, in the dually challenged group that received DFM in their diet (DSP group), the frequency of lungs exhibiting lung consolidation was also reduced (*P* < 0.05). Thus, the provision of DFM in the diet resulted in a significant decrease in extent and severity of gross lung pathology compared to the case with the identically challenged pigs that did not receive DFM.

**TABLE 2:** Experimental design to test the effect of DFM provision on a Salmonella/PRRSV coinfection (DFM effect trial)

Group no.	Group designation	No. of pigs/group	Treatment designation	Treatment day			
				−21	0	3	8/9
1	NMM	4	No DFM, mock challenge		Mock	Mock	Euthanasia
2	NSM	11	No DFM, Salmonella challenge, mock virus challenge		Salmonella	Mock	Euthanasia
3	DSM	11	DFM, Salmonella challenge, mock virus challenge	Start DFM	Salmonella	Mock	Euthanasia
4	NSP	11	No DFM, Salmonella challenge, PRRSV challenge		Salmonella	PRRSV	Euthanasia
5	DSP	11	DFM, Salmonella challenge, PRRSV challenge	Start DFM	Salmonella	PRRSV	Euthanasia

### Dual-pathogen-challenged pigs receiving DFM exhibited a reduction in the rate of Salmonella lung colonization and decreased load of PRRSV in the lung.

**FIG 3:**
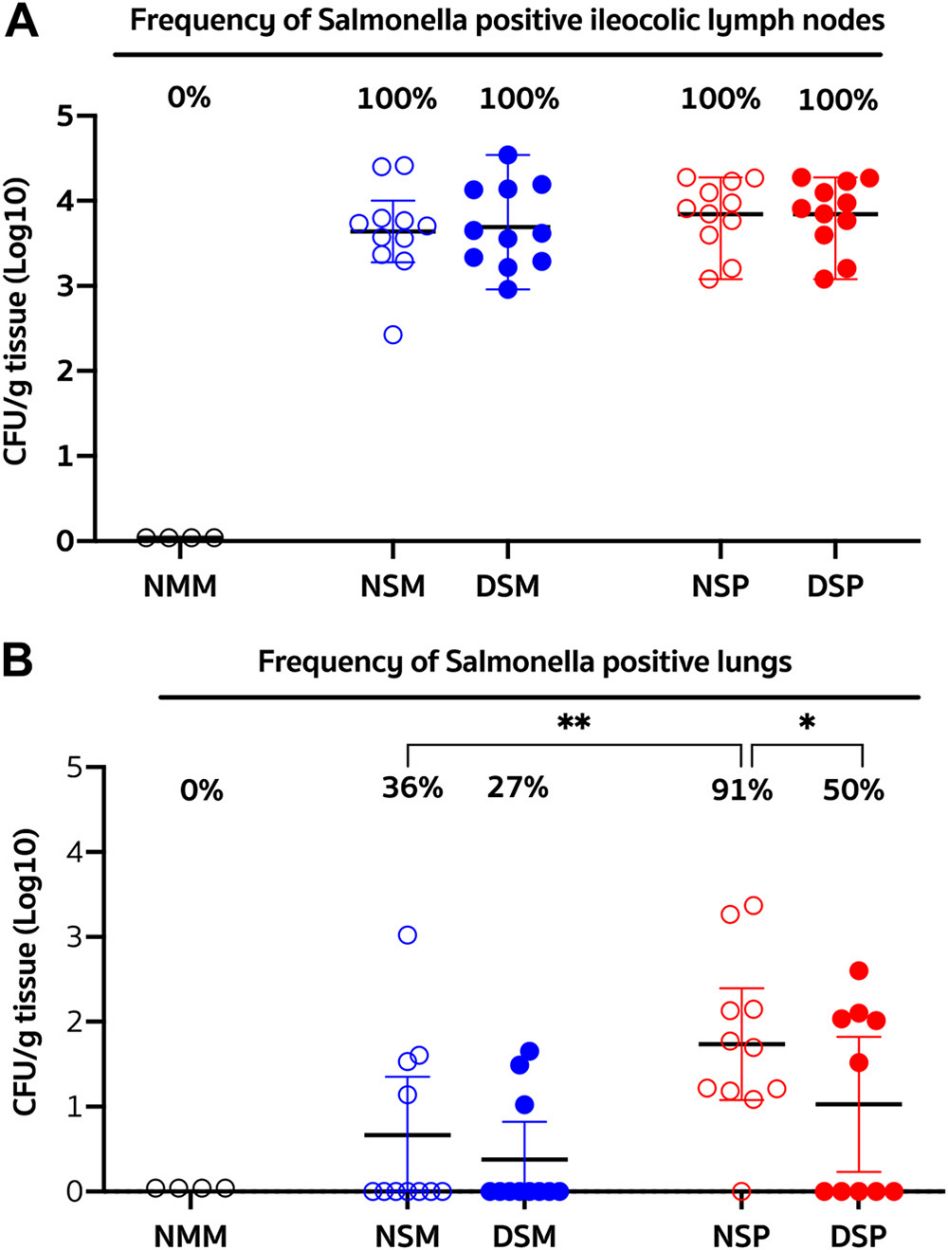
Extent and frequency of Salmonella colonization of the ileocolic lymph node and lung. Weaner pigs were fed for 21 days with a standard nursery diet supplemented with DFM (filled circles) or without supplementation (empty circles). Afterwards, the pigs were challenged via gavage with Salmonella enterica serotype Choleraesuis, followed 3 days later with an intranasal challenge with either PRRSV (red circles) or a mock inoculum (blue circles). A control group of animals (*n* = 4) which were fed a nonsupplemented diet served as the strict control (black circles). Each symbol represents the number of Salmonella CFU per gram of tissue per pig grouped by treatment, and each group is identified by its corresponding abbreviation as listed for Fig. 2. The graph shows the mean and 95% confidence limits for each group, and, at the top, the corresponding frequency (%) of Salmonella-positive ileocecal lymph nodes (A) or lungs (B). Differences between groups in the frequency of Salmonella-positive ileocecal lymph nodes were analyzed using Barnard’s exact test. Differences were considered significant if the *P* value was ≤0.05. *, *P* < 0.05; **, *P* < 0.01.

**FIG 4:**
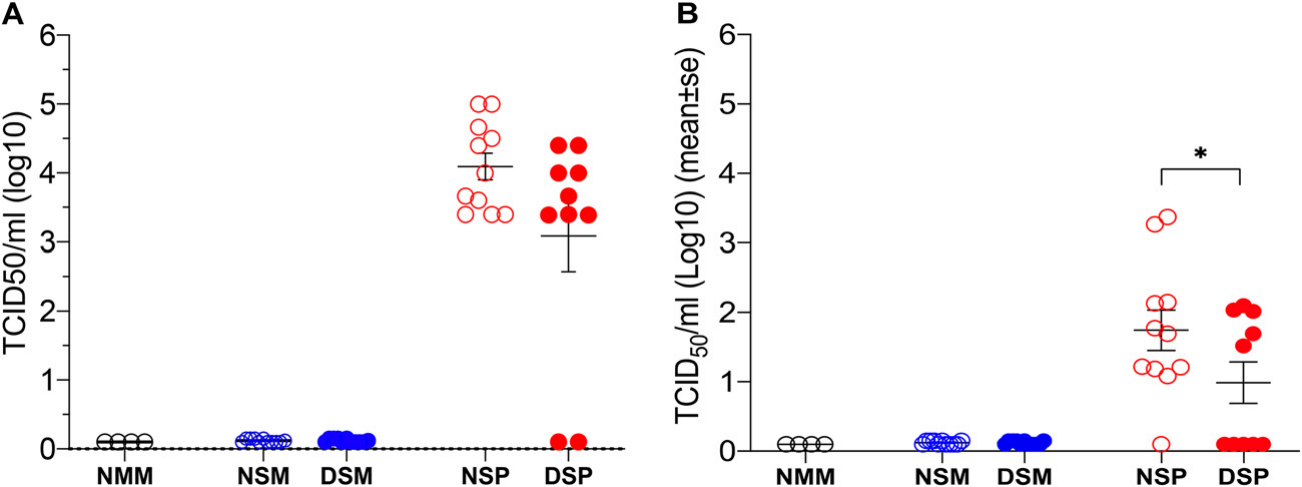
Extent and frequency of viremia and virus load in the lung. The amount of infectious virus in serum (A) and bronchoalveolar lavage (BAL) fluid (B) samples collected at the time of necropsy was determined. The titer of infectious virus is expressed as 50% tissue culture infectious dose (TCID_50_). Each symbol represents sample results from a single pig grouped according to their treatment, and each group is identified by its corresponding abbreviation as listed for Fig. 2. Horizontal bars represent the mean ± SE for each group. Statistically significant difference between the identically challenged groups was determined using the Mann-Whitney test. A *P* value of ≤0.05 was considered significant. *, *P* < 0.05.

Regardless of DFM treatment, Salmonella organisms were isolated in similar quantities from the ICLN collected from each member in all four groups challenged with S. Choleraesuis (Fig. 3A). Efforts to isolate Salmonella from the respiratory tract (Fig. 3B) resulted in a 36% rate of isolation from the lungs of pigs challenged only with Salmonella (NSM group), which is similar to the 27% rate of isolation in pigs supplemented with DFM (DSM group). In contrast, the rate of Salmonella isolation from the lungs of dually challenged pigs that did not receive DFM in their diet (NSP group) was 91%, while in the identically challenged pigs that received DFM (DSP group), the rate of isolation was reduced to 50% (*P* < 0.05). Examination of the virus load in serum of dually challenged pigs administered DFM revealed a 10-fold reduction in the group’s mean level of viremia compared to that of the identically challenged group that did not receive DFM (Fig. 4A). Although this reduction did not reach statistical significance, two of the pigs treated with DFM exhibited no detectable viremia, and the level of viremia in six other pigs in this group was below the mean viremia exhibited by the non-DFM-treated pigs. Notably, only 5 of 10 pigs in the DFM-treated group had detectable virus in bronchoalveolar (BAL) fluids harvested at the time of necropsy, while 10 of 11 pigs in the non-DFM-treated group had virus present, resulting in a significant difference (*P* < 0.05) in the pulmonary viral loads between these two groups (Fig. 4B).

### Pigs receiving DFM exhibited an increased expression of NOD2 and TREM-1 after pathogen challenge.

**FIG 5:**
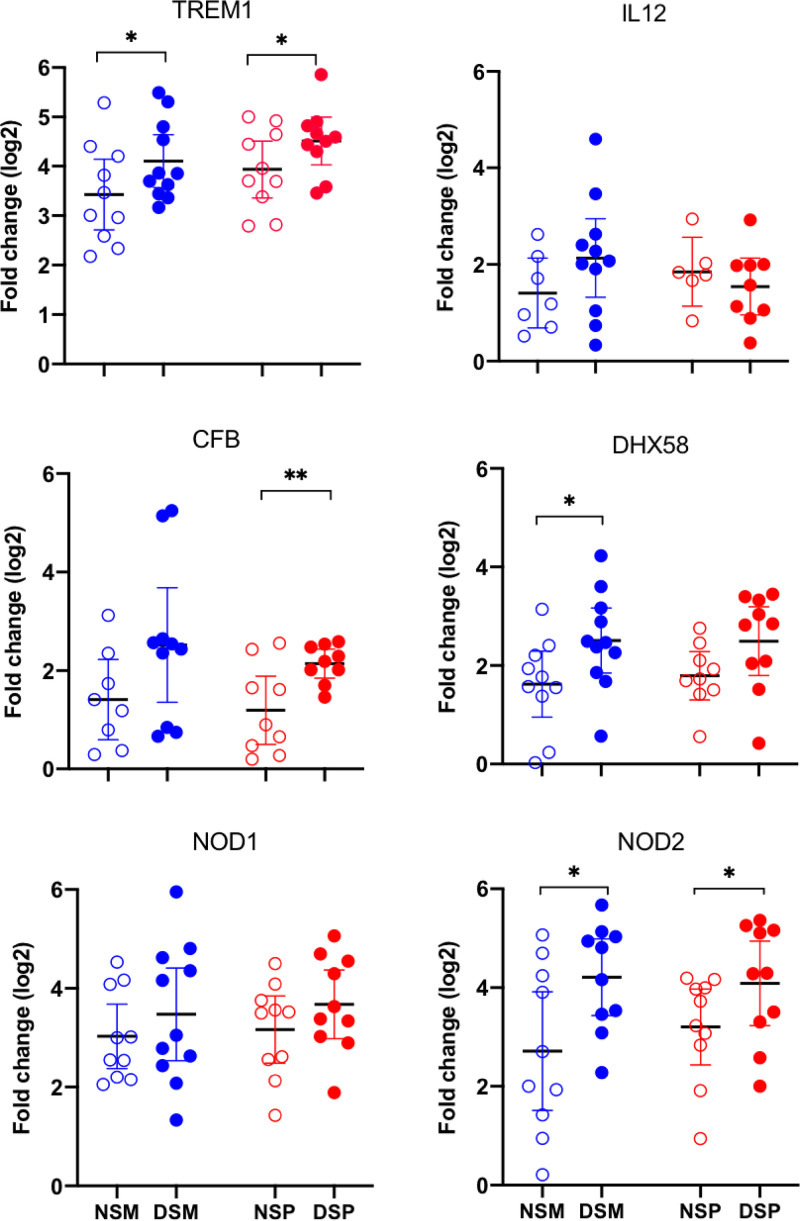
Changes in immune-related gene expression in whole blood. Changes in the extent of gene expression in blood collected at the time of necropsy were determined using real-time reverse transcription-PCR, using the comparative cycle threshold (*C_T_*) method and the formula 2^−ΔΔ^*^CT^* (76), where the GAPDH gene was used as the reference housekeeping gene. All values are expressed as fold change relative to control. Each symbol represents sample results from a single pig grouped according to their treatment, and each group is identified by its corresponding abbreviation as listed for Fig. 2. The graphs show the mean and 95% confidence limits for each group. The effect of DFM supplementation between identically challenged groups was analyzed by unpaired *t* test using data normalized by log_2_ transformation. A *P* value of <0.05 was considered significant. *, *P* < 0.05; **, *P* < 0.01.

Differential expression of immune-related genes after the pathogen challenge was assessed in whole-blood samples collected at the time of necropsy. Among 54 immune-related genes examined, 6 exhibited distinguishable differential expression between the identically challenged groups that received and did not receive DFM in their diet (Fig. 5). A statistically significant (*P* < 0.05) increased expression of genes encoding triggering receptor expressed on myeloid cells 1 (TREM-1) and the nucleotide-binding oligomerization domain 2 (NOD2) receptor was found in the DFM-treated groups, compared to the identically challenged non-DFM-treated group (Fig. 5). Expression for the complement factor B (CFB) protein and the signaling molecule DExH-box helicase 58 (DHX58), also known as LGP2, trended upwards in DFM-treated animals compared to the non-DFM-treated group. However, the CFB gene reached statistical significance (*P* < 0.05) only in the groups challenged with both Salmonella and PRRSV. Conversely, DHX58 reached statistical significance between the groups challenged only with Salmonella. In the DFM-treated groups, NOD1 showed trends for increased gene expression but did not reach statistical significance.

### Dual-pathogen-challenged pigs treated with DFM exhibited enhanced levels of IL-1 and IL-8 in their lungs.

**FIG 6:**
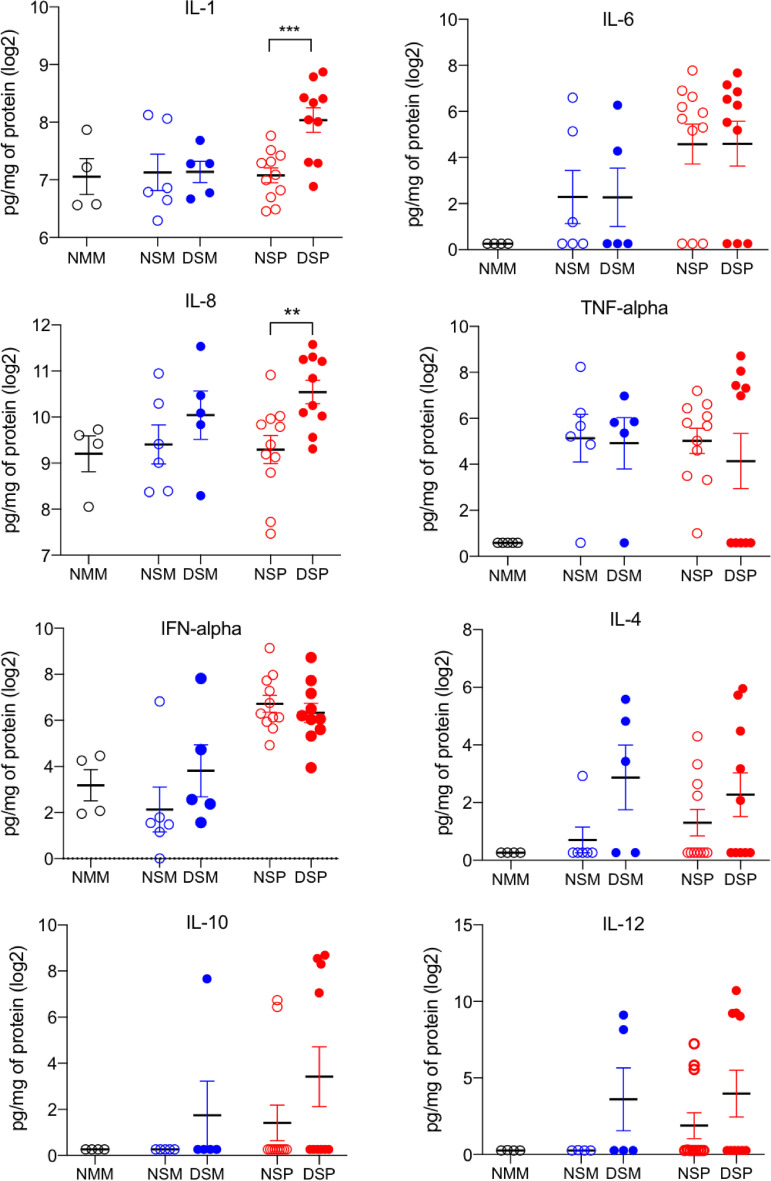
Proinflammatory (IL-1, IL-6, IL-8, and TNF-α) and immunoregulatory (IFN-α, IL-4, IL-10, and IL-12) cytokines in BAL fluid. Bronchoalveolar lavage was performed in the lungs harvested from animals at the time of necropsy. Cytokine levels in BAL fluid were measured using a porcine cytokine bead array. Each symbol represents sample results from a single pig grouped according to their treatment, and each treatment group is identified by its corresponding abbreviation as listed for Fig. 2. Horizontal bars represent the mean ± SE for each group. The level of cytokine measured was adjusted to the total amount of protein present in the sample and is reported as picogram of cytokine per 1 mg/mL of protein. Cytokine levels were compared between the identically challenged DFM treated and nontreated groups using a one-tailed Mann-Whitney test with log_2_ transformation.

Levels of proinflammatory (interleukin 1 [IL-1], IL-6, IL-8, and tumor necrosis factor alpha [TNF-α]) and immunoregulatory (alpha interferon [IFN-α], IFN-γ, IL-4, IL-10, and IL-12) cytokines were evaluated in BAL samples collected at the time of necropsy. There were no significant differences in cytokine levels between the Salmonella-challenged groups regardless of DFM treatment (Fig. 6). The two groups challenged with both Salmonella and PRRSV, regardless of DFM treatment, exhibited an increase in IFN-α compared to that in the mock-treated group, which is consistent with reports showing that infection with PRRSV triggers a transient increase of this cytokine in BAL fluid (31, 32). However, compared to the dually challenged non-DFM-treated group, the DFM-treated group exhibited a significant increase in IL-1 (*P* < 0.001) and IL-8 (*P* < 0.01) levels (Fig. 6). The enhanced presence of IL-1 and IL-8 in the lung suggests that the provision of DFM strengthened the response of these two cytokines when confronted with the dual bacterial/viral challenge.

## DISCUSSION

Among the nontyphoidal Salmonella serotypes, S. Choleraesuis is the most invasive to the host, capable of spreading systemically and colonizing multiple organs (24, 25, 33, 34). Typical gross lesions observed in pigs afflicted with a systemic S. Choleraesuis infection include enterocolitis, enlarged mesenteric lymph nodes, randomly distributed pale foci in the liver, and diffuse interstitial pneumonia of various intensities (24, 26, 28, 29). In this study, the experimental infection of weanling pigs with S. Choleraesuis resulted in the development of all of these pathological changes. The observed 100% colonization rate of the ILCN by Salmonella and its sporadic spread to the lung is consistent with its described pathogenesis (23–25, 35–37). This study also reproduced previous observations that coinfection of weanling pigs with S. Choleraesuis and PRRSV results in a synergistic syndrome (27, 28). This syndrome was exhibited by an exacerbated pneumonia characterized by an increase in the extent of lung involvement and lung consolidation. Notably, histological analyses revealed that the exacerbated interstitial pneumonia, characterized grossly by lung tissue consolidation, was the result of heightened infiltration of the alveolar septum by mononuclear cells resulting in a marked reduction of air space. The synergistic syndrome was also manifested by a higher rate of Salmonella isolation from the lungs of pigs challenged with both Salmonella and PRRSV without DFM treatment than for the group challenged with only S. Choleraesuis. This observation suggests that the viral infection somehow enhanced the bacteria’s ability to spread systemically and colonize the lung. Although the exact route by which S. Choleraesuis achieves extraintestinal infection in swine has not been determined, the persistence of S. Choleraesuis in mesenteric lymph nodes is associated with its ability to produce systemic infection (38). A proposed model for the extraintestinal dissemination of S. enterica serotype Typhimurium consists of gastrointestinally derived CD18-expressing mononuclear phagocytes transporting the bacteria via the bloodstream to other organs (39, 40). Consistent with this model is the presence of mononuclear phagocytes in the capillaries of a pig’s lung carrying S. Choleraesuis in their phagosomes (21), thus pointing to monocytes as players in the systemic spread of S. Choleraesuis in swine. Under normal baseline conditions, resident lung macrophages are replaced by monocytes with low kinetics, whereas during an acute inflammatory response, there is a brisk acceleration of this process (41). PRRSV replicates primarily in lung macrophages (42), and although it does not patently replicate in monocytes, pulmonary infection with PRRSV triggers a large influx of monocytes into the lung (31). Once there, migrating blood monocytes give rise to lung macrophages (43) and become targets for PRRSV infection (32, 44). Although without direct evidence, it is tempting to speculate that the observed increased Salmonella lung colonization rate in pigs coinfected with S. Choleraesuis and PRRSV is driven by the virus infection-induced influx of Salmonella-infected monocytes into the lungs. In addition, Salmonella has recently been shown to exploit the unfolded protein response (UPR) to promote intracellular replication (45), and the ability of invasive Salmonella enterica to trigger apoptosis in infected macrophage is a mechanism that has been proposed to facilitate its systemic spread (33, 34). The replication of PRRSV in macrophages produces endoplasmic reticulum (ER) stress, which triggers the UPR and ultimately results in apoptosis (46). Thus, another mechanism that might explain the enhanced rate of lung colonization by Salmonella could be a higher rate of bacterial replication in virus-infected macrophages promoted by the UPR response of virus-infected cells and their apoptotic death. Future studies will be directed at exploring these hypotheses.

Our results demonstrated that the provision of the Bacillus subtilis-based DFM, for 3 weeks prior to an S. Choleraesuis challenge, reduced the extent of pneumonia caused by this pathogen. These results are consistent with previous reports showing that while probiotics are not able to affect the rate of colonization of mesenteric lymph nodes by Salmonella enterica (47), they can ameliorate the inflammatory syndrome (48, 49) and the systemic spread of Salmonella (12, 50). Provision of DFM also mitigated the pathogenic synergy resulting from the S. Choleraesuis and PRRSV coinfection, evidenced by a reduced extent and severity of interstitial pneumonia, as well as a reduced rate of Salmonella lung colonization. However, since the provision of DFM did not affect the rate or extent of Salmonella colonization of the ILCN in these animals, it is likely that the reduced systemic spread of the bacteria in the dually challenged pigs treated with DFM resulted from a mitigating effect on events occurring after this step in the pathogenesis of the synergistic syndrome. Because DFM provision reduced the rate of Salmonella lung colonization in the dually challenged group but not in the group challenged only with Salmonella, we speculate that the DFM treatment mitigated an event triggered by the virus infection which resulted in a higher bacterial presence in the lung. The reduced viral load in the lung provides evidence suggesting that the DFM elicited an antiviral innate immune function distal to the gastrointestinal tract.

The increased expression of NOD2 and TREM-1 in the blood of Salmonella-challenged DFM-treated pigs provides evidence in support of a systemic effect by the DFM. Expression of NOD2 is upregulated in monocytes and macrophages after exposure to bacterial wall components such as lipopolysaccharide (LPS), lipoteichoic acid (LTA), and peptidoglycan (PGN) degradation products (51, 52). The NOD2 receptor is localized in the cytosol of monocytes, macrophages, and granulocytes, where it senses muramyl dipeptide (MDP), a degradation product of PGN (53, 54). Both endosomal trafficking and outer membrane vesicular transport have been proposed as suitable methods for shuttling PGN to enable extracellular sensing of this bacterial molecule by cytosolic NOD-like receptors (55, 56). The existence of this and other pathways capable of delivering fragments of PGN into the cytosol has been proposed as a mechanism by which NOD receptors are able to sense ligands released by extracellular bacteria, including those derived from the gut microbiota (57, 58). The gut microbiota has been shown to mediate protection against pneumococcal pneumonia by enhancing alveolar phagocytic activity as well as proinflammatory cytokine secretion (59). Systemic distribution of PGN translocated from the gut was shown to enhance neutrophil function in the bone marrow, and this effect was positively correlated with PGN levels in serum (60). A growing body of evidence indicates that once in the blood, systemic PGN plays an important role in regulating myeloid cell homeostasis and modulating the maturation and antimicrobial function of phagocytes (58, 61). The NOD2 ligand MDP has been shown to enhance innate immunity against Klebsiella pneumoniae respiratory infection by enhancing alveolar macrophage reactive oxygen species (ROS)-mediated bacterial killing (17). Since NOD2 also functions as a cytoplasmic sensor for viral single-stranded RNA (ssRNA), NOD2 is now considered to play a major role in host defense against both viral and bacterial pathogens affecting the lung (62). For instance, the provision of MDP to mice challenged with influenza virus induced a NOD2-dependent recruitment of inflammatory monocytes and neutrophils into the lung and provided protection against influenza virus infection (63). Previous data have shown that LTA, a Toll-like receptor 2 (TLR-2) ligand and component of the Gram-positive bacterial cell wall, induces the expression of TREM-1 through a MyD88-dependent pathway (64, 65). Translocation of LTA into the blood has been documented to occur as a result of having a microbiota enriched with bacteria of the *Firmicutes* phylum (66). TREM-1 is a receptor expressed on neutrophils and monocytes that amplifies the inflammatory response to microbial products, effectively lowering the threshold of pathogen detection (67). TREM-1 signaling enhances the expression of NOD2 in blood mononuclear cells, resulting in an increased sensitivity of NOD2 to low concentrations of MDP (65). TREM-1 activation triggers the release of IL-8 from neutrophils and monocytes, and the release of IL-8 and TNF-α by monocytes is strongly upregulated by priming with LPS (68). Similarly, activation of TREM-1 enhances production of IL-1β, IL-6, and TNF-α in response to the NOD2 ligand MDP (65).

Elevated levels of IL-1 and IL-8 in the lungs of dually challenged DFM-treated animals suggest that innate cells mediated a stronger response when confronted with the microbial challenge. This interpretation is consistent with the notion that a more robust antibacterial and antiviral immunity in the lung can be driven by the intestinal microbiome. Although the mechanisms underlying the protection against systemic salmonellosis in pigs are ill defined, it is known that protection from S. enterica is associated with the IL-8-mediated induction of polymorphonucleated cells (69). In addition, genetic traits associated with resistance to S. Choleraesuis in swine include a more efficient phagocytosis and killing of Salmonella by neutrophils (70). Furthermore, high levels of IL-1 and IL-8 in serum have been linked to PRRSV clearance (71).

The contribution of the intestinal microbiome in pulmonary host defense is an emerging concept offering great promise for the development of strategies to protect against respiratory infections (16, 18, 72, 73). Among the four major bacterial phyla that comprise the mammalian microbiota, members of *Firmicutes* phylum of the *Bacilli* class are the strongest NOD2 stimulators (74). The DFM used in this study, Provent ECL, is comprised of spores from several proprietary strains of Bacillus subtilis, which belong to this class. Therefore, we hypothesize the observed reduced rate of Salmonella lung colonization in dually challenged DFM-treated animals might be related to the lung’s macrophages having been instructed by products derived from the DFM to mediate a more effective antiviral immune response and thus better able to resist viral infection. This assumption is supported by data showing the absence of virus in the lungs of 50% of the DFM-treated animals. Reduced viral replication in the lung would be expected to reduce the influx of Salmonella-infected monocytes to this organ triggered by the presence of the virus (31), which, in turn, could mitigate Salmonella’s ability to successfully colonize the lung. Our results are consistent with the report that DFM comprised of a consortium of *Firmicutes* bacteria can regulate and enhance respiratory immunity and promote resistance to lung infection by bacteria through NOD2 (74). We speculate that the increased expression of NOD2 and TREM1 in the blood of DFM-treated pigs could be attributed to cell wall components, namely, PGN and LTA of the Bacillus subtilis strains comprising Provent ECL. Alternatively, it is also possible that DFM strains compete with preexisting flora, leading to the lysis and solubilization of cellular components from themselves or other Gram-positive cells (75), which could result in their absorption and systemic distribution.

The data presented are consistent with the notion that the Bacillus subtilis based-DFM Provent ECL can modulate cells of the innate immune system systemically, resulting in an enhanced protective immunity against a microbial challenge. Our results are also consistent with the concept that low-level tonic stimulation by microbial products poises innate immune cells to fight viral infections (16, 76, 77). The provision of DFM could be acting in a way similar to what has been described for bacillus Calmette-Guérin (BCG) vaccination, in which the induction of cytokines via NOD2 signaling enhances innate immune defenses mediated by monocytes (78). This innate immune enhancing effect by BCG is termed “trained immunity” and has been shown to strengthen innate immune defenses against viral infections (79, 80). Future studies will be aimed at deciphering the mechanism by which the Bacillus subtilis-based DFM promotes the development of protective innate immune defenses of the respiratory tract.

## MATERIALS AND METHODS

### Animals, diets, housing, and experimental design.

Two animal trials were conducted. The first trial was aimed at experimentally confirming the reported pathogenic synergy between S. Choleraesuis and PRRSV (28). The second trial was aimed at assessing the prophylactic effect of DFM on mitigating the ill effects of a respiratory infectious disease. Five-week-old mixed-breed pigs were sourced from a swine research farm at the University of Illinois, free of PRRSV and *Mycoplasma*. Piglets tested negative for Salmonella spp. at the start of the study as determined by testing fecal swabs using bacteriologic culture with selective enrichment. For each study, the animals were housed in a suite at the biosafety level 2 (BSL2) biocontainment facility at the University of Illinois at Urbana-Champaign (UIUC). Each treatment group was housed in a separate, environmentally isolated pen within the suite. For the Salmonella/PRRSV synergy trial, eight pigs were allocated to three treatment groups. Two groups (*n* = 3) were inoculated with 10^8^ CFU of S. Choleraesuis delivered into the esophagus, followed 3 days later with an intranasal administration with either 5 × 10^4^ 50% tissue culture infectious doses (TCID_50_) of PRRSV strain NADC20 (challenge) or a mock inoculum. The bacterial challenge was administered using gelatin capsules deposited into the esophagus using a pill dispenser. Pigs in the third group (*n* = 2) received a capsule with a mock inoculum and were challenged 3 days later with PRRSV. The animals were monitored for 9 days after the bacterial challenge, euthanized, and necropsied. For the DFM effect trial, 48 pigs were randomly assigned to the five treatment groups listed in Table 2. The pigs were transitioned to a standard balanced soybean/corn swine phase 2 nursery diet, with (D) or without (N) 3-lb/ton supplementation of Provent ECL (lot CX0020180321). Provent ECL is a direct-fed microbial product comprising six Bacillus subtilis strains applied at an inclusion of 3.67 × 10^5^ CFU/g of feed. *Bacillus* strains were delivered as spores in calcium carbonate, maltodextrin, and mineral oil carriers. Three weeks later, animals in groups 2, 3, 4, and 5 were challenged with 10^9^ CFU of Salmonella enterica serotype Choleraesuis (S). To ensure a consistent level of infection, a 10-fold-higher dose was used than in the initial experiment. The bacterial challenge was administered into the esophagus using a pill dispenser containing gelatin capsules loaded with the bacteria. The treatment groups for this experiment are listed in Table 2. Pigs in group 1 received a gelatin capsule with a mock inoculum (M). Three days later, animals in groups 4 and 5 were intranasally challenged with 5 × 10^4^ TCID_50_ of PRRSV strain NADC20 (P) and pigs in groups 1, 2, and 3 received a mock virus inoculum (M). After the challenge, animals were monitored daily for 8 to 9 days for the presence of clinical signs, including changes in vitality (depression), dyspnea, coughing, chills, vomiting, and/or diarrhea. For logistical purposes, half of the animals in each of the treatment groups were euthanized 8 days after the bacterial challenge and the other half the following day. Following euthanasia, a necropsy was performed, and various tissue samples were collected for subsequent bacteriologic, virologic, and molecular assays.

### Salmonella inoculum preparation.

Salmonella enterica serovar Choleraesuis strain 4271GB was kindly provided by Eric Vimr (81). Confirmation that the 4271GB isolate belongs to the Salmonella O antigen C1 serogroup (Choleraesuis) and is a Kunzendorf variant was done by serological testing at the National Veterinary Services Laboratory (Ames, IA). The 4271GB strain produced black colonies in xylose-Tergitol-lysine 4 (XLT4) agar plates (Remel Products), indicating its ability to produce hydrogen sulfide. A pure colony of 4271GB was added to 30 mL of Mueller-Hinton (MH) broth (BD Diagnostics, Sparks, MD) and cultured for 15 h at 37°C. The concentration of bacteria present in the culture broth was determined by measuring absorbance at 600 nm using a NanoDrop One instrument (Thermo Fisher) following the manufacturer’s instructions and using samples serially diluted to be within the linear range of the optical system. Based on the measurement of the optical density at 600 nm (OD_600_), the culture was concentrated to contain 1 × 10^9^ bacteria in 0.25 mL and loaded into gelatin capsules with approximately 0.75 g of feed as previously described (35). The capsule was inserted past the larynx into the proximal end of the esophagus by use of a pill dispenser. The actual viable cell count data were confirmed by plating 10-fold serial dilutions of the inoculum onto XLT4 agar plates (Remel Products). Based on the viable count, the dose administered to the animals was 2 × 10^9^ CFU.

### Virus inoculum preparation.

The “atypical PRRS abortion storm” virus isolate NADC-20 (82) was passaged once in the porcine alveolar macrophage cell line ZMAC to create the challenge virus stock as previously described (83). Briefly, a cell-free preparation of the virus was prepared by centrifuging the medium overlaying the infected cell monolayers at 4°C and 350 × *g* for 10 min. Aliquots of the virus were stored at −80°C, and a sample of the virus stock was titrated for the presence of infectious virus. The virus titer was calculated using the Reed and Muench method (84) and expressed as TCID_50_ per milliliter.

### Gross and microscopic pathological examination of lungs and other organs at necropsy.

Necropsies were performed by a pathologist, and the presence of lesions in the thoracic and abdominal viscera was recorded. The extent of gross lung pathology was assessed by calculating a mean percentage value of the lung exhibiting gross visible pneumonia (e.g., tan mottled areas and areas of consolidation) based on the percentage of each lobe affected according to the previously described standard scoring method (85). The extent of tan mottled areas and areas of consolidation were recorded separately. Lung sections were taken for histopathological examination. Blood and serum samples, lung tissue, and bronchial and ileocecal lymph node tissues were collected for bacteriologic examination. Bronchoalveolar lavage (BAL) fluid was collected as previously described (86) and tested for viral load and cytokine content.

### Salmonella detection and quantification in tissues.

Lymph nodes and lung tissue were harvested at the time of necropsy and placed on ice immediately after collection. Samples were transferred to the laboratory for processing within 2 to 5 h after collection and were macerated in LB medium using a stomacher. The resulting macerate was clarified by gravity sedimentation and 10-fold serial dilutions prepared in LB media. A 0.15-mL volume of each dilution was plated in duplicate onto XLT4 agar, distributed onto the medium surface with a sterile L-shaped bacterial spreader, and incubated at 35°C. Black colonies with appearance characteristic of Salmonella were counted after 2 days of incubation. The total CFU obtained per milliliter was adjusted to CFU per gram according to the dilution of the inoculum that yielded a countable range of black colonies and the weight of the tissue used to prepare the macerate.

### Determination of viremia and viral load in the lung.

Serum and BAL samples collected at the time of necropsy were used to measure viral load. The titer of infectious virus in these samples was determined as previously described (87). Briefly, a 0.1-mL volume of 10-fold serial dilutions of the samples were plated into flat-bottom 96-well tissue culture plates. Immediately afterwards, 0.1 mL of ZMAC cells at 4 × 10^5^ cells/mL was added to each well as the cell substrate for virus replication. Virus titers were calculated and expressed as TCID_50_ per milliliter by the Reed and Muench calculation method (84).

### Measurement of pulmonary cytokines.

Serum and BAL samples obtained at the time of necropsy were tested for the presence of the cytokines IL-1β, IL-4, IL-6, IL-8, IL-10, IFN-α, IFN-γ, and TNF-α using a Milliplex porcine cytokine/chemokine magnetic bead panel multiplex assay (Millipore) and analyzed using a Bio-Rad Luminex cytometry bead analyzer. In BAL samples, the cytokine concentration was adjusted to the total amount of protein in the sample. Total protein content was determined by quantitative colorimetry using total protein reagent (Sigma; product number T 1949) following the manufacturer’s recommended procedure.

### Assessment of the expression of immune cell markers in whole-blood samples.

On the day of necropsy, whole blood was collected in Tempus blood RNA tubes (Thermo Fisher), and RNA was extracted using the MagMax kit for stabilized blood tubes (Thermo Fisher) following the manufacturer’s protocol. Immune marker gene expression was measured using TaqMan real-time PCR assays (Thermo Fisher) and the quantitative PCR (qPCR) system as previously described (46). The porcine genes examined were those encoding CTLA4, ITGAM, NOS2, glyceraldehyde-3-phosphate dehydrogenase (GAPDH), ACTB, FoxP3, CXCL2, IFN-β, Casp8, TLR-2, IL-2RA, IL-10, CD19, IL-6, SOCS2, NOD2, SOCS3, GRN, STAT3, NF-κB1, TLR-3, CFB, LY96, SELL, TLR-4, NOD1, IFN-γ, IL-12, IL-18, C3, TNF, CD8a, CD4, NF-κBIA, CXCL12, SIRPA, TREM-1, IL-2, CXCL8, TLR-9, CD163, IL-1B, JAK2, IL-4, CD40, IFN-α, IL-17F, TLR-10, CD209, GZMB, DHX58, DDX58, MS4A1, and TGFB1. The Thermo Fisher assay identifier (ID) for each of these genes is listed in Table S1 in the supplemental material. The resulting data were analyzed using the cycle threshold (2^−ΔΔ^*^CT^*) method, with data normalized to the reference GAPDH gene (88). All values are expressed as fold change relative to the control.

### Statistical analysis.

Differences in the extent of gross lung pathology were analyzed using one-way analysis of variance (ANOVA), followed by Tukey’s multiple comparisons. Differences between groups in the number of CFU per gram of tissue were determined by comparing identically challenged groups via Welch’s *t* test. Differences in the frequency distribution of lungs showing any extent of consolidation, as well as the rate of Salmonella-positive tissues, were analyzed using Barnard’s exact test. Cytokine levels, as well as viral load in serum and BAL samples, were compared between the identically challenged DFM-treated and nontreated groups using a one-tailed Mann-Whitney test. Differences in gene expression between identically challenged groups were determined by unpaired *t* test using log_2_-transformed data. Statistical analyses were performed using GraphPad Prism 8.4.2 (San Diego, CA), except for Barnard’s exact test, which was done using In-Silico Online (89). Differences where the *P* value was <0.05 were considered significant.

### Ethical approval.

This study was approved by the University of Illinois Institutional Animal Care and Usage Committee (protocol number 19048) and was conducted in compliance with local and federal guidelines regulating laboratory animal care and housing.

## References

[1] Whittemore CT, Green DM. 2001. Growth of the young weaned pig, p 1–15. In Varley MA, Wiseman J (ed), The weaner pig: nutrition and management. Proceedings of a British Society of Animal Science Occasional Meeting, University of Nottingham, UK, September 2000. CABI, Wallingford, United Kingdom.

[2] Willing BP, Malik G, Van Kessel AG. 2013. Nutrition and gut health in swine, p 197–213. *In* Chiba LI (ed), Sustainable swine nutrition. John Wiley & Sons, Ltd, Chichester, United Kingdom.

[3] Varley MA, Wiseman J. 2001. The weaner pig: nutrition and management. Proceedings of a British Society of Animal Science Occasional Meeting, University of Nottingham, UK, September 2000. CABI, Wallingford, United Kingdom.

[4] Pluske JR, Turpin DL, Kim J-C. 2018. Gastrointestinal tract (gut) health in the young pig. Anim Nutr 4:187–196. 10.1016/j.aninu.2017.12.004. 30140758 PMC6104527

[5] Lallès J-P, Bosi P, Smidt H, Stokes CR. 2007. Nutritional management of gut health in pigs around weaning. Proc Nutr Soc 66:260–268. 10.1017/S0029665107005484. 17466106

[6] Xiong X, Tan B, Song M, Ji P, Kim K, Yin Y, Liu Y. 2019. Nutritional intervention for the intestinal development and health of weaned pigs. Front Vet Sci 6:46. 10.3389/fvets.2019.00046. 30847348 PMC6393345

[7] Thaiss CA, Zmora N, Levy M, Elinav E. 2016. The microbiome and innate immunity. Nature 535:65–74. 10.1038/nature18847. 27383981

[8] Libertucci J, Young VB. 2019. The role of the microbiota in infectious diseases. Nat Microbiol 4:35–45. 10.1038/s41564-018-0278-4. 30546094

[9] Tran THT, Everaert N, Bindelle J. 2018. Review on the effects of potential prebiotics on controlling intestinal enteropathogens Salmonella and Escherichia coli in pig production. J Anim Physiol Anim Nutr (Berl) 102:17–32. 10.1111/jpn.12666. 28028851

[10] Fouhse JM, Zijlstra RT, Willing BP. 2016. The role of gut microbiota in the health and disease of pigs. Anim Front 6:30–36. 10.2527/af.2016-0031.

[11] Sanders ME, Merenstein DJ, Reid G, Gibson GR, Rastall RA. 2019. Probiotics and prebiotics in intestinal health and disease: from biology to the clinic. Nat Rev Gastroenterol Hepatol 16:605–616. 10.1038/s41575-019-0173-3. 31296969

[12] Splichal I, Donovan SM, Splichalova Z, Neuzil Bunesova V, Vlkova E, Jenistova V, Killer J, Svejstil R, Skrivanova E, Splichalova A. 2019. Colonization of germ-free piglets with commensal Lactobacillus amylovorus, Lactobacillus mucosae, and probiotic E. coli Nissle 1917 and their interference with Salmonella Typhimurium. Microorganisms 7:273. 10.3390/microorganisms7080273. PMC6722580 31434337

[13] Mingmongkolchai S, Panbangred W. 2018. Bacillus probiotics: an alternative to antibiotics for livestock production. J Appl Microbiol 124:1334–1346. 10.1111/jam.13690. 29316021

[14] Caulier S, Nannan C, Gillis A, Licciardi F, Bragard C, Mahillon J. 2019. Overview of the antimicrobial compounds produced by members of the Bacillus subtilis group. Front Microbiol 10:302. 10.3389/fmicb.2019.00302. 30873135 PMC6401651

[15] Zhang W, Zhu Y-H, Zhou D, Wu Q, Song D, Dicksved J, Wang J-F. 2017. Oral administration of a select mixture of Bacillus probiotics affects the gut microbiota and goblet cell function following Escherichia coli challenge in newly weaned pigs of genotype MUC4 that are supposed to be enterotoxigenic E. coli F4ab/ac receptor negative. Appl Environ Microbiol 83:e02747-16. 10.1128/AEM.02747-16. 27881419 PMC5244294

[16] McAleer JP, Kolls JK. 2018. Contributions of the intestinal microbiome in lung immunity. Eur J Immunol 48:39–49. 10.1002/eji.201646721. 28776643 PMC5762407

[17] Clarke TB. 2014. Early innate immunity to bacterial infection in the lung is regulated systemically by the commensal microbiota via nod-like receptor ligands. Infect Immun 82:4596–4606. 10.1128/IAI.02212-14. 25135683 PMC4249320

[18] Budden KF, Gellatly SL, Wood DLA, Cooper MA, Morrison M, Hugenholtz P, Hansbro PM. 2017. Emerging pathogenic links between microbiota and the gut-lung axis. Nat Rev Microbiol 15:55–63. 10.1038/nrmicro.2016.142. 27694885

[19] Wang Y, Gao L, Zhang Y, Shi C, Ren C. 2014. Efficacy of probiotic therapy in full-term infants with critical illness. Asia Pac J Clin Nutr 23:575–580. 25516315 10.6133/apjcn.2014.23.4.14

[20] Gelardi M, La Mantia I, Drago L, Meroni G, Aragona SE, Cupido G, Vicini C, Berardi C, Ciprandi G, Italian Study Group on Upper Respiratory Infections, et al. 2020. A probiotic mixture in patients with upper respiratory diseases: the point of view of the otorhinolaringologist. J Biol Regul Homeost Agents 34:5–10. 33426860

[21] Baskerville A, Dow C, Curran WL, Hanna J. 1972. Ultrastructure of phagocytosis of Salmonella cholerae-suis by pulmonary macrophages in vivo. Br J Exp Pathol 53:641–647. 4646199 PMC2072486

[22] Ibrahim H, Askar B, Hulme S, Neilson P, Barrow P, Foster N. 2018. Differential immune phenotypes in human monocytes induced by non-host-adapted Salmonella enterica serovar Choleraesuis and host-adapted S. Typhimurium. Infect Immun 86:e00509-18. 10.1128/IAI.00509-18. 30037797 PMC6204746

[23] Anderson RC, Nisbet DJ, Buckley SA, Genovese KJ, Harvey RB, Deloach JR, Keith NK, Stanker LH. 1998. Experimental and natural infection of early weaned pigs with Salmonella choleraesuis. Res Vet Sci 64:261–262. 10.1016/s0034-5288(98)90136-9. 9690614

[24] Reed WM, Olander HJ, Thacker HL. 1986. Studies on the pathogenesis of Salmonella typhimurium and Salmonella choleraesuis var kunzendorf infection in weanling pigs. Am J Vet Res 47:75–83. 3511804

[25] Watson PR, Paulin SM, Jones PW, Wallis TS. 2000. Interaction of Salmonella serotypes with porcine macrophages in vitro does not correlate with virulence. Microbiology 146:1639–1649. 10.1099/00221287-146-7-1639. 10878128

[26] Kolb J, Hoffman L. 1990. Salmonella choleraesuis as a cause of respiratory disease in growing and finishing swine. Iowa State Univ Vet 52:66–69.

[27] Stevenson GW, Van Alstine WG, Kanitz CL, Keffaber KK. 1993. Endemic porcine reproductive and respiratory syndrome virus infection of nursery pigs in two swine herds without current reproductive failure. J Vet Diagn Invest 5:432–434. 10.1177/104063879300500322. 8373860

[28] Wills RW, Gray JT, Fedorka-Cray PJ, Yoon KJ, Ladely S, Zimmerman JJ. 2000. Synergism between porcine reproductive and respiratory syndrome virus (PRRSV) and Salmonella choleraesuis in swine. Vet Microbiol 71:177–192. 10.1016/S0378-1135(99)00175-3. 10703702 PMC7117217

[29] Lawson GH, Dow C. 1966. Porcine salmonellosis. A study of the field disease. J Comp Pathol 76:363–371. 10.1016/0021-9975(66)90056-9. 6008378

[30] Jubb KVF, Kennedy PC. 1970. Pathology of domestic animals, 2nd ed. Academic Press, New York, NY.

[31] Renson P, Rose N, Le Dimna M, Mahé S, Keranflec’h A, Paboeuf F, Belloc C, Le Potier M-F, Bourry O. 2017. Dynamic changes in bronchoalveolar macrophages and cytokines during infection of pigs with a highly or low pathogenic genotype 1 PRRSV strain. Vet Res 48:15. 10.1186/s13567-017-0420-y. 28241868 PMC5327547

[32] Nazki S, Khatun A, Jeong C-G, Mattoo SUS, Gu S, Lee S-I, Kim S-C, Park J-H, Yang M-S, Kim B, Park C-K, Lee S-M, Kim W-I. 2020. Evaluation of local and systemic immune responses in pigs experimentally challenged with porcine reproductive and respiratory syndrome virus. Vet Res 51:66. 10.1186/s13567-020-00789-7. 32404209 PMC7222343

[33] Guiney DG, Fang FC, Krause M, Libby S, Buchmeier NA, Fierer J. 1995. Biology and clinical significance of virulence plasmids in Salmonella serovars. Clin Infect Dis 21(Suppl 2):S146–S151. 10.1093/clinids/21.supplement_2.s146. 8845442

[34] Guiney DG, Fierer J. 2011. The role of the spv genes in Salmonella pathogenesis. Front Microbiol 2:129. 10.3389/fmicb.2011.00129. 21716657 PMC3117207

[35] Gray JT, Fedorka-Cray PJ, Stabel TJ, Ackermann MR. 1995. Influence of inoculation route on the carrier state of Salmonella choleraesuis in swine. Vet Microbiol 47:43–59. 10.1016/0378-1135(95)00060-n. 8604554

[36] Uthe JJ, Royaee A, Lunney JK, Stabel TJ, Zhao S-H, Tuggle CK, Bearson SMD. 2007. Porcine differential gene expression in response to Salmonella enterica serovars Choleraesuis and Typhimurium. Mol Immunol 44:2900–2914. 10.1016/j.molimm.2007.01.016. 17337057

[37] Gray JT, Fedorka-Cray PJ, Stabel TJ, Kramer TT. 1996. Natural transmission of Salmonella choleraesuis in swine. Appl Environ Microbiol 62:141–146. 10.1128/aem.62.1.141-146.1996. 8572691 PMC167782

[38] Paulin SM, Jagannathan A, Campbell J, Wallis TS, Stevens MP. 2007. Net replication of Salmonella enterica serovars Typhimurium and Choleraesuis in porcine intestinal mucosa and nodes is associated with their differential virulence. Infect Immun 75:3950–3960. 10.1128/IAI.00366-07. 17548482 PMC1952012

[39] Vazquez-Torres A, Jones-Carson J, Bäumler AJ, Falkow S, Valdivia R, Brown W, Le M, Berggren R, Parks WT, Fang FC. 1999. Extraintestinal dissemination of Salmonella by CD18-expressing phagocytes. Nature 401:804–808. 10.1038/44593. 10548107

[40] Vazquez-Torres A, Fang FC. 2000. Cellular routes of invasion by enteropathogens. Curr Opin Microbiol 3:54–59. 10.1016/s1369-5274(99)00051-x. 10679413

[41] Maus UA, Janzen S, Wall G, Srivastava M, Blackwell TS, Christman JW, Seeger W, Welte T, Lohmeyer J. 2006. Resident alveolar macrophages are replaced by recruited monocytes in response to endotoxin-induced lung inflammation. Am J Respir Cell Mol Biol 35:227–235. 10.1165/rcmb.2005-0241OC. 16543608

[42] Duan X, Nauwynck HJ, Pensaert MB. 1997. Effects of origin and state of differentiation and activation of monocytes/macrophages on their susceptibility to porcine reproductive and respiratory syndrome virus (PRRSV). Arch Virol 142:2483–2497. 10.1007/s007050050256. 9672608 PMC7086874

[43] Evren E, Ringqvist E, Tripathi KP, Sleiers N, Rives IC, Alisjahbana A, Gao Y, Sarhan D, Halle T, Sorini C, Lepzien R, Marquardt N, Michaëlsson J, Smed-Sörensen A, Botling J, Karlsson MCI, Villablanca EJ, Willinger T. 2021. Distinct developmental pathways from blood monocytes generate human lung macrophage diversity. Immunity 54:259–275.e7. 10.1016/j.immuni.2020.12.003. 33382972

[44] Gómez-Laguna J, Salguero FJ, Barranco I, Pallarés FJ, Rodríguez-Gómez IM, Bernabé A, Carrasco L. 2010. Cytokine expression by macrophages in the lung of pigs infected with the porcine reproductive and respiratory syndrome virus. J Comp Pathol 142:51–60. 10.1016/j.jcpa.2009.07.004. 19691969 PMC7126906

[45] Antoniou AN, Lenart I, Kriston-Vizi J, Iwawaki T, Turmaine M, McHugh K, Ali S, Blake N, Bowness P, Bajaj-Elliott M, Gould K, Nesbeth D, Powis SJ. 2019. Salmonella exploits HLA-B27 and host unfolded protein responses to promote intracellular replication. Ann Rheum Dis 78:74–82. 10.1136/annrheumdis-2018-213532. 30355574 PMC6317449

[46] Chen W-Y, Schniztlein WM, Calzada-Nova G, Zuckermann FA. 2018. Genotype 2 strains of porcine reproductive and respiratory syndrome virus dysregulate alveolar macrophage cytokine production via the unfolded protein response. J Virol 92:e01251-17. 10.1128/JVI.01251-17. PMC5752938 29070690

[47] Walsh MC, Rostagno MH, Gardiner GE, Sutton AL, Richert BT, Radcliffe JS. 2012. Controlling Salmonella infection in weanling pigs through water delivery of direct-fed microbials or organic acids: part II. Effects on intestinal histology and active nutrient transport. J Anim Sci 90:2599–2608. 10.2527/jas.2010-3599. 22344321

[48] Yang G-Y, Yu J, Su J-H, Jiao L-G, Liu X, Zhu Y-H. 2017. Oral administration of Lactobacillus rhamnosus GG ameliorates Salmonella Infantis-induced inflammation in a pig model via activation of the IL-22BP/IL-22/STAT3 pathway. Front Cell Infect Microbiol 7:323. 10.3389/fcimb.2017.00323. 28770173 PMC5514694

[49] Liu X, Xia B, He T, Li D, Su J-H, Guo L, Wang J-F, Zhu Y-H. 2019. Oral administration of a select mixture of Lactobacillus and Bacillus alleviates inflammation and maintains mucosal barrier integrity in the ileum of pigs challenged with Salmonella Infantis. Microorganisms 7:135. 10.3390/microorganisms7050135. PMC6560431 31096680

[50] Splichalova A, Jenistova V, Splichalova Z, Splichal I. 2019. Colonization of preterm gnotobiotic piglets with probiotic Lactobacillus rhamnosus GG and its interference with Salmonella Typhimurium. Clin Exp Immunol 195:381–394. 10.1111/cei.13236. 30422309 PMC6378394

[51] Gutierrez O, Pipaon C, Inohara N, Fontalba A, Ogura Y, Prosper F, Nunez G, Fernandez-Luna JL. 2002. Induction of Nod2 in myelomonocytic and intestinal epithelial cells via nuclear factor-kappa B activation. J Biol Chem 277:41701–41705. 10.1074/jbc.M206473200. 12194982

[52] Takahashi Y, Isuzugawa K, Murase Y, Imai M, Yamamoto S, Iizuka M, Akira S, Bahr GM, Momotani E-I, Hori M, Ozaki H, Imakawa K. 2006. Up-regulation of NOD1 and NOD2 through TLR4 and TNF-alpha in LPS-treated murine macrophages. J Vet Med Sci 68:471–478. 10.1292/jvms.68.471. 16757890

[53] Girardin SE, Boneca IG, Viala J, Chamaillard M, Labigne A, Thomas G, Philpott DJ, Sansonetti PJ. 2003. Nod2 is a general sensor of peptidoglycan through muramyl dipeptide (MDP) detection. J Biol Chem 278:8869–8872. 10.1074/jbc.C200651200. 12527755

[54] Girardin SE, Travassos LH, Hervé M, Blanot D, Boneca IG, Philpott DJ, Sansonetti PJ, Mengin-Lecreulx D. 2003. Peptidoglycan molecular requirements allowing detection by Nod1 and Nod2. J Biol Chem 278:41702–41708. 10.1074/jbc.M307198200. 12871942

[55] Irving AT, Mimuro H, Kufer TA, Lo C, Wheeler R, Turner LJ, Thomas BJ, Malosse C, Gantier MP, Casillas LN, Votta BJ, Bertin J, Boneca IG, Sasakawa C, Philpott DJ, Ferrero RL, Kaparakis-Liaskos M. 2014. The immune receptor NOD1 and kinase RIP2 interact with bacterial peptidoglycan on early endosomes to promote autophagy and inflammatory signaling. Cell Host Microbe 15:623–635. 10.1016/j.chom.2014.04.001. 24746552

[56] Nakamura N, Lill JR, Phung Q, Jiang Z, Bakalarski C, de Mazière A, Klumperman J, Schlatter M, Delamarre L, Mellman I. 2014. Endosomes are specialized platforms for bacterial sensing and NOD2 signalling. Nature 509:240–244. 10.1038/nature13133. 24695226

[57] Philpott DJ, Sorbara MT, Robertson SJ, Croitoru K, Girardin SE. 2014. NOD proteins: regulators of inflammation in health and disease. Nat Rev Immunol 14:9–23. 10.1038/nri3565. 24336102

[58] Bastos PAD, Wheeler R, Boneca IG. 2021. Uptake, recognition and responses to peptidoglycan in the mammalian host. FEMS Microbiol Rev 45:fuaa044. 10.1093/femsre/fuaa044. 32897324 PMC7794044

[59] Schuijt TJ, Lankelma JM, Scicluna BP, de Sousa e Melo F, Roelofs JJTH, de Boer JD, Hoogendijk AJ, de Beer R, de Vos A, Belzer C, de Vos WM, van der Poll T, Wiersinga WJ. 2016. The gut microbiota plays a protective role in the host defence against pneumococcal pneumonia. Gut 65:575–583. 10.1136/gutjnl-2015-309728. 26511795 PMC4819612

[60] Clarke TB, Davis KM, Lysenko ES, Zhou AY, Yu Y, Weiser JN. 2010. Recognition of peptidoglycan from the microbiota by Nod1 enhances systemic innate immunity. Nat Med 16:228–231. 10.1038/nm.2087. 20081863 PMC4497535

[61] Wolf AJ, Underhill DM. 2018. Peptidoglycan recognition by the innate immune system. Nat Rev Immunol 18:243–254. 10.1038/nri.2017.136. 29292393

[62] Wiese KM, Coates BM, Ridge KM. 2017. The role of nucleotide-binding oligomerization domain-like receptors in pulmonary infection. Am J Respir Cell Mol Biol 57:151–161. 10.1165/rcmb.2016-0375TR. 28157451 PMC5576584

[63] Coulombe F, Fiola S, Akira S, Cormier Y, Gosselin J. 2012. Muramyl dipeptide induces NOD2-dependent Ly6C(high) monocyte recruitment to the lungs and protects against influenza virus infection. PLoS One 7:e36734. 10.1371/journal.pone.0036734. 22590599 PMC3348889

[64] Zheng H, Heiderscheidt CA, Joo M, Gao X, Knezevic N, Mehta D, Sadikot RT. 2010. MYD88-dependent and -independent activation of TREM-1 via specific TLR ligands. Eur J Immunol 40:162–171. 10.1002/eji.200839156. 19904768

[65] Netea MG, Azam T, Ferwerda G, Girardin SE, Kim S-H, Dinarello CA. 2006. Triggering receptor expressed on myeloid cells-1 (TREM-1) amplifies the signals induced by the NACHT-LRR (NLR) pattern recognition receptors. J Leukoc Biol 80:1454–1461. 10.1189/jlb.1205758. 16940328

[66] Loo TM, Kamachi F, Watanabe Y, Yoshimoto S, Kanda H, Arai Y, Nakajima-Takagi Y, Iwama A, Koga T, Sugimoto Y, Ozawa T, Nakamura M, Kumagai M, Watashi K, Taketo MM, Aoki T, Narumiya S, Oshima M, Arita M, Hara E, Ohtani N. 2017. Gut microbiota promotes obesity-associated liver cancer through PGE2-mediated suppression of antitumor immunity. Cancer Discov 7:522–538. 10.1158/2159-8290.CD-16-0932. 28202625

[67] Klesney-Tait J, Turnbull IR, Colonna M. 2006. The TREM receptor family and signal integration. Nat Immunol 7:1266–1273. 10.1038/ni1411. 17110943

[68] Bouchon A, Dietrich J, Colonna M. 2000. Cutting edge: inflammatory responses can be triggered by TREM-1, a novel receptor expressed on neutrophils and monocytes. J Immunol 164:4991–4995. 10.4049/jimmunol.164.10.4991. 10799849

[69] Foster N, Lovell MA, Marston KL, Hulme SD, Frost AJ, Bland P, Barrow PA. 2003. Rapid protection of gnotobiotic pigs against experimental salmonellosis following induction of polymorphonuclear leukocytes by avirulent Salmonella enterica. Infect Immun 71:2182–2191. 10.1128/IAI.71.4.2182-2191.2003. 12654840 PMC152035

[70] van Diemen PM, Kreukniet MB, Galina L, Bumstead N, Wallis TS. 2002. Characterisation of a resource population of pigs screened for resistance to salmonellosis. Vet Immunol Immunopathol 88:183–196. 10.1016/s0165-2427(02)00165-4. 12127416

[71] Lunney JK, Fritz ER, Reecy JM, Kuhar D, Prucnal E, Molina R, Christopher-Hennings J, Zimmerman J, Rowland RRR. 2010. Interleukin-8, interleukin-1beta, and interferon-gamma levels are linked to PRRS virus clearance. Viral Immunol 23:127–134. 10.1089/vim.2009.0087. 20373993

[72] Wypych TP, Wickramasinghe LC, Marsland BJ. 2019. The influence of the microbiome on respiratory health. Nat Immunol 20:1279–1290. 10.1038/s41590-019-0451-9. 31501577

[73] Winkler ES, Thackray LB. 2019. A long-distance relationship: the commensal gut microbiota and systemic viruses. Curr Opin Virol 37:44–51. 10.1016/j.coviro.2019.05.009. 31226645 PMC6768733

[74] Brown RL, Sequeira RP, Clarke TB. 2017. The microbiota protects against respiratory infection via GM-CSF signaling. Nat Commun 8:1512. 10.1038/s41467-017-01803-x. 29142211 PMC5688119

[75] Adams CA. 2010. The probiotic paradox: live and dead cells are biological response modifiers. Nutr Res Rev 23:37–46. 10.1017/S0954422410000090. 20403231

[76] Abt MC, Artis D. 2013. The dynamic influence of commensal bacteria on the immune response to pathogens. Curr Opin Microbiol 16:4–9. 10.1016/j.mib.2012.12.002. 23332724 PMC3622187

[77] McAleer JP, Kolls JK. 2012. Maintaining poise: commensal microbiota calibrate interferon responses. Immunity 37:10–12. 10.1016/j.immuni.2012.07.001. 22840839

[78] Kleinnijenhuis J, Quintin J, Preijers F, Joosten LAB, Ifrim DC, Saeed S, Jacobs C, van Loenhout J, de Jong D, Stunnenberg HG, Xavier RJ, van der Meer JWM, van Crevel R, Netea MG. 2012. Bacille Calmette-Guerin induces NOD2-dependent nonspecific protection from reinfection via epigenetic reprogramming of monocytes. Proc Natl Acad Sci USA 109:17537–17542. 10.1073/pnas.1202870109. 22988082 PMC3491454

[79] Arts RJW, Moorlag S, Novakovic B, Li Y, Wang S-Y, Oosting M, Kumar V, Xavier RJ, Wijmenga C, Joosten LAB, Reusken CBEM, Benn CS, Aaby P, Koopmans MP, Stunnenberg HG, van Crevel R, Netea MG. 2018. BCG vaccination protects against experimental viral infection in humans through the induction of cytokines associated with trained immunity. Cell Host Microbe 23:89–100.e5. 10.1016/j.chom.2017.12.010. 29324233

[80] Moorlag S, Arts RJW, van Crevel R, Netea MG. 2019. Non-specific effects of BCG vaccine on viral infections. Clin Microbiol Infect 25:1473–1478. 10.1016/j.cmi.2019.04.020. 31055165

[81] Lichtensteiger CA, Vimr ER. 2003. Systemic and enteric colonization of pigs by a hilA signature-tagged mutant of Salmonella choleraesuis. Microb Pathog 34:149–154. 10.1016/s0882-4010(02)00196-1. 12631476

[82] Harms PA, Sorden SD, Halbur PG, Bolin SR, Lager KM, Morozov I, Paul PS. 2001. Experimental reproduction of severe disease in CD/CD pigs concurrently infected with type 2 porcine circovirus and porcine reproductive and respiratory syndrome virus. Vet Pathol 38:528–539. 10.1354/vp.38-5-528. 11572560

[83] Calzada-Nova G, Schnitzlein WM, Husmann RJ, Zuckermann FA. 2011. North American porcine reproductive and respiratory syndrome viruses inhibit type I interferon production by plasmacytoid dendritic cells. J Virol 85:2703–2713. 10.1128/JVI.01616-10. 21191013 PMC3067927

[84] Reed LJ, Muench H. 1938. A simple method of estimating fifty per cent endpoints. Am J Epidemiol 27:493–497. 10.1093/oxfordjournals.aje.a118408.

[85] Halbur PG, Paul PS, Frey ML, Landgraf J, Eernisse K, Meng XJ, Lum MA, Andrews JJ, Rathje JA. 1995. Comparison of the pathogenicity of two US porcine reproductive and respiratory syndrome virus isolates with that of the Lelystad virus. Vet Pathol 32:648–660. 10.1177/030098589503200606. 8592800

[86] Thomas DJ, Husmann RJ, Villamar M, Winship TR, Buck RH, Zuckermann FA. 2011. Lactobacillus rhamnosus HN001 attenuates allergy development in a pig model. PLoS One 6:e16577. 10.1371/journal.pone.0016577. 21386995 PMC3046142

[87] Meier WA, Galeota J, Osorio FA, Husmann RJ, Schnitzlein WM, Zuckermann FA. 2003. Gradual development of the interferon-gamma response of swine to porcine reproductive and respiratory syndrome virus infection or vaccination. Virology 309:18–31. 10.1016/S0042-6822(03)00009-6. 12726723

[88] Livak KJ, Schmittgen TD. 2001. Analysis of relative gene expression data using real-time quantitative PCR and the 2(−Delta Delta C(T)) method. Methods 25:402–408. 10.1006/meth.2001.1262. 11846609

[89] Joosse SA. In-Silico Online. http://in-silico.online/. Accessed 28 February 2021.

