# Supplementary material for: Bacillus-Based Direct-Fed Microbial Reduces the Pathogenic Synergy of a Coinfection with Salmonella enterica Serovar Choleraesuis and Porcine Reproductive and Respiratory Syndrome Virus
Source: Infect Immun. 2022 Mar 7;90(4):e00574-21. doi: 10.1128/iai.00574-21 (PMC9022502; doi:10.1128/iai.00574-21)
Supplement: Supplemental file 1 — Supplemental material. Download iai.00574-21-s0001.pdf, PDF file, 0.06 MB [file iai.00574-21-s0001.pdf]

Supplementary Table 1. Thermo Fisher TaqMan Assay ID of the porcine genes examined.

Thermo Fisher Assay ID	Gene Symbol
Ss03394068_m1	JAK2
Ss04248268_m1	DHX58
Ss03385661_u1	SOCS2
Ss03391255_m1	C3
Ss03388869_m1	C6
Ss03389383_g1	CFB
Ss03374608_u1	NOS2
Ss03388413_m1	GRN
Ss03820946_s1	GZMB
Ss03393804_m1	IL-1B
Ss03376563_uH	ACTB
Ss03381754_u1	IL2RA
Ss03379427_u1	CASP8
Ss03392428_m1	IL-2
Ss03391054_m1	IFN-γ
Ss03381278_u1	TLR2
Ss03394125_m1	IL-4
Ss03394862_g1	IFN-α
Ss03388861_m1	TLR3
Ss03384604_u1	IL-6
Ss03378485_u1	IFN-β
Ss03389780_m1	TLR4
Ss03391176_m1	IL-12
Ss03392437_m1	CXCL8
Ss03393060_u1	TLR9
Ss03382372_u1	IL-10
Ss03378360_u1	CXCL2
Ss03375629_u1	GAPDH
Ss03391203_m1	IL-18
Ss03391855_m1	CXCL12
Ss03388575_m1	NFκB1
Ss03391318_g1	TNF
Ss03391676_m1	CD4
Ss03388426_m1	STAT3
Ss03395044_gH	IL17F
Ss03373919_m1	IL22
Ss04955543_m1	TGFB1
Ss03391669_m1	CD8A
Ss03391861_m1	SIRPA

Ss03384432_u1	CD19
Ss03374588_m1	ITGAM
Ss03376695_u1	FOXP3
Ss04324123_m1	MS4A1
Ss03392220_m1	TREM1
Ss03393162_u1	CD163
Ss03819234_g1	CD209
Ss03394337_m1	CD40
Ss03213761_m1	CTLA4
Ss03389733_m1	SELL
Ss03818641_s1	TLR10
Ss03391764_m1	NFKBIA
Ss03389452_m1	LY96
Ss03389985_m1	NOD1
Ss03386634_u1	NOD2
Ss04322983_m1	DDX58
Ss03387992_u1	SOCS3
